# SuboptimalDietaryPatterns and Estimated Cardiovascular Disease Mortality Burden in China: A Repeated Cross-Sectional Comparative Risk Assessment

**DOI:** 10.3390/nu18183043

**Published:** 2026-09-17

**Authors:** Meng-Jie He, Lichun Huang, Peiwei Xu, Danting Su, Yan Zou, Dan Han, Gangqiang Ding, Ronghua Zhang, Pengyu Fu

**Affiliations:** 1 National Institute for Nutrition and Health, Chinese Center for Disease Control and Prevention, 29 Nanwei Road, Beijing 100050, China; mjhe@cdc.zj.cn; 2 Department of Nutrition and Food Safety, Zhejiang Provincial Center for Disease Control and Prevention, Hangzhou 310051, China; lchuang@cdc.zj.cn (L.H.); pwxu@cdc.zj.cn (P.X.); dtsu@cdc.zj.cn (D.S.); yzou@cdc.zj.cn (Y.Z.); dhan@cdc.zj.cn (D.H.); 3 NHC Specialty Laboratory of Food Safety Risk Assessment and Standard Development, Hangzhou 310051, China; 4 Public Health Institute, Henan Provincial Center for Disease Control and Prevention, Zhengzhou 450016, China

**Keywords:** suboptimal dietary factors, cardiovascular disease, population-attributable fraction, Jiangnan diet

## Abstract

**Background/Objectives**: Suboptimal diet is a major modifiable contributor to cardiovascular disease (CVD) mortality in China, and provincial disparities in dietary quality may contribute to geographic variation in CVD burden. This study aimed to quantify temporal trends in CVD mortality attributable to suboptimal dietary factors in Zhejiang Province and evaluate the potential reduction in CVD deaths in Henan Province under a counterfactual Jiangnan dietary pattern. **Methods:** This repeated cross-sectional study used data from multiple rounds of population-based nutrition surveillance conducted in Zhejiang Province between 2008 and 2024, with additional comparative data from Henan Province in 2015. A total of 56,018 adults were included. Dietary intake was assessed using 3-day 24-h dietary recalls combined with household condiment weighing. Ten dietary factors were evaluated, including fruits, vegetables, aquatic products, nuts, whole grains, red meat, processed meat, sugar-sweetened beverages, condiment salt, and omega-3 fatty acids. The prevalence of suboptimal dietary exposures was standardized according to the urban–rural population distribution. Population-attributable fractions (PAFs) of cardiovascular disease (CVD) mortality associated with individual and combined dietary factors were estimated using a comparative risk-assessment framework based on published relative-risk estimates. A counterfactual scenario was further used to estimate the number of CVD deaths in Henan Province that might be averted if the diet-attributable PAF were reduced to the level observed in coastal Zhejiang. **Results**: The dietary profile in Zhejiang was characterized by relatively lower prevalences of inadequate intake of aquatic products, whole grains, and vegetables, together with a decline in excessive salt intake. The estimated proportion of CVD mortality attributable to suboptimal dietary factors based on comparative risk assessment was 49.8% (95% UI, 40.8–57.4) in 2008, increased numerically to 55.3% (48.1–61.6) in 2022, and was 52.5% (45.6–58.6) in 2024. Among the examined subgroups, women living in coastal Zhejiang had the lowest estimated diet-attributable PAF. Zhejiang had a healthier dietary profile and a lower PAF for diet-attributable CVD mortality than Henan. Under the specified counterfactual scenario, by matching Henan’s dietary pattern to that of coastal Zhejiang, it was estimated that approximately 23,575 annual CVD deaths could be averted. **Conclusions:** Suboptimal dietary exposures remained responsible for a substantial and persistent proportion of CVD mortality. Although Zhejiang had a lower estimated diet-attributable CVD mortality burden than Henan, important gaps remained, particularly for low intakes of nuts, fruit, and whole grains. Refining the Jiangnan dietary pattern and adapting its favorable components to high-burden regions may inform region-specific CVD prevention.

## 1. Introduction

Cardiovascular diseases (CVDs) are the leading cause of death globally, accounting for an estimated 17.9 million deaths annually and more than one-third of all deaths worldwide [1,2]. The burden is particularly severe in China, where an estimated 330 million people have CVD and approximately 40% of all deaths are attributable to CVD conditions [3]. Population ageing, the increasing prevalence of hypertension, diabetes, and obesity, and persistent unhealthy lifestyles are expected to further increase CVD incidence and mortality rates in China are expected to rise [4,5,6]. However, many of these lifestyle-associated risks are modifiable.

Among the modifiable CVD risk factors, diet plays a central role [7]. Suboptimal dietary patterns, characterized by excessive intake of foods associated with higher CVD risk and insufficient intake of protective foods, contribute substantially to cardiometabolic diseases [8,9]. These patterns largely reflect the progressing trend toward Westernization of diets in China and the consequent rise in the proportion of dietary energy derived from fats, along with structural imbalances, such as high salt intake, insufficient consumption of fruits and vegetables, and low whole-grain intake [10,11]. These changes in the dietary structure of Chinese residents have provided a scientific basis for research on the population-attributable fraction (PAF) of CVDs to suboptimal diets, which can support the development of dietary improvement policies and health economic evaluations. However, although one previous population-based cross-sectional study used the PAF to quantify the impact of suboptimal dietary factors on the CVD mortality burden among Chinese adults [12], the currently available research has not incorporated the latest data from the past 15 years.

**Figure 1:**
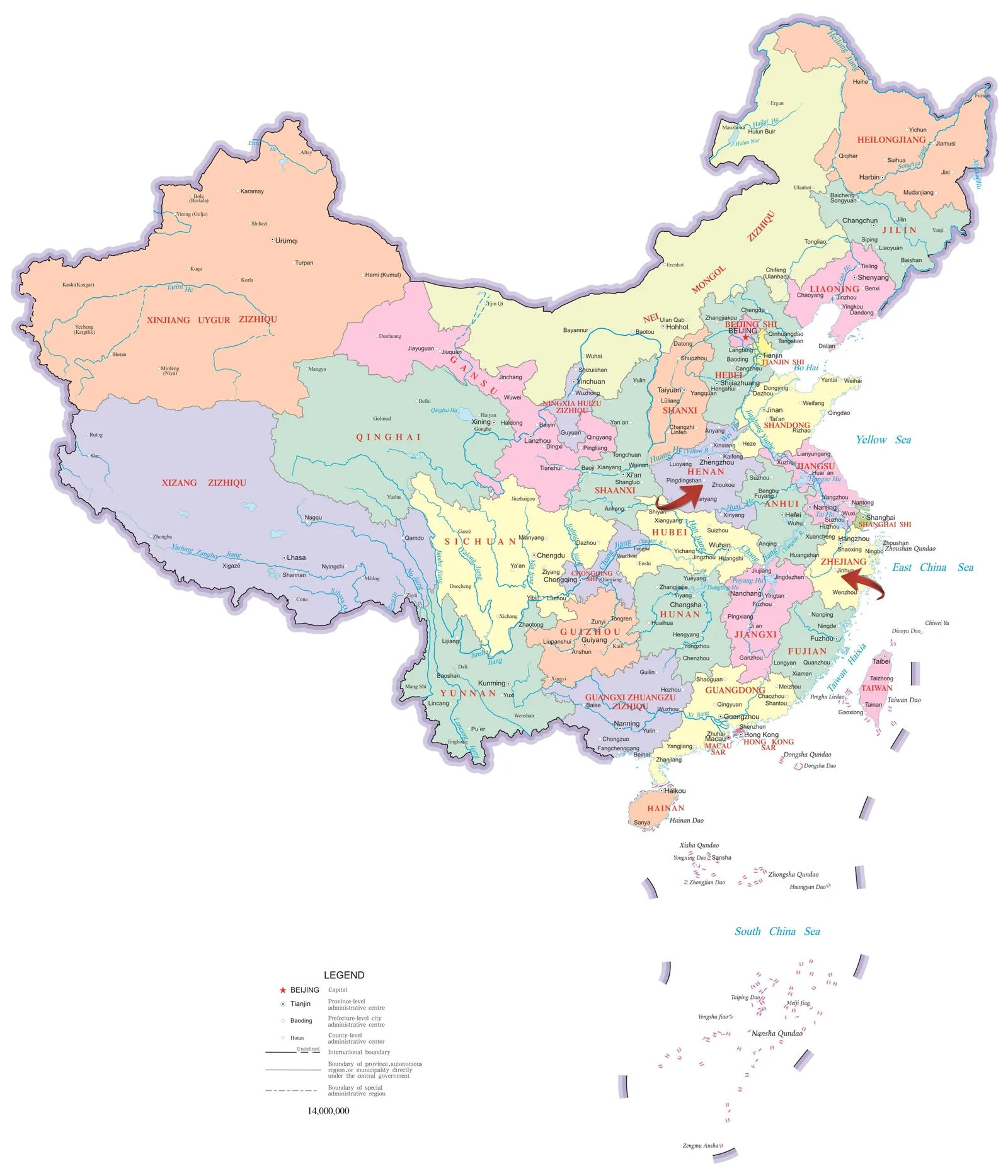
Mapofthe study areas. The map was obtained from the Standard Map Service System of the Ministry of Natural Resources of the People’s Republic of China (Review No.: GS (2022) 4310). The red arrows indicate Henan Province and Zhejiang Province, the study regions included in this research.

More importantly, comparative analyses between specific regions are still lacking, despite the recognition that distinct dietary patterns characterize different regions in China [13,14]. For example, Zhejiang Province, located in the southern Yangtze River Delta (Figure 1), has both coastal and inland influences and has traditionally adhered to the Jiangnan diet [14,15,16], which is characterized by mild use of salt and seasonings and an abundant use of aquatic products. However, economic development has led to increased consumption of sweets [17,18], preserved foods, and processed items high in oil and salt [19,20]. This has created a unique dietary profile in which both traditional protective factors and newly introduced risk factors for CVD coexist, making Zhejiang an ideal region for examining the diet–CVD relationship.

As a counterpoint, Henan Province, located in central China (Figure 1), shares historical and cultural connections with Zhejiang, partly reflecting population migration during the Southern Song Dynasty (1127–1279). Nevertheless, the two provinces differ considerably in socioeconomic development, healthcare access, urbanization, lifestyle, and other determinants of cardiovascular health. In Henan Province, CVD is the leading factor threatening residents’ health. The 2025 Henan Report on Cardiovascular Health and Diseases [21] indicates that both the incidence and mortality rates of CVD have been on a steady upward trend, with Henan ranking second in CVD mortality in China. Cardiovascular and cerebrovascular diseases account for nearly 60% of all deaths in Henan [22], and the annual average growth rate of CVD mortality among registered residents in Henan increased to 6.83% between 2018 and 2022. Thus, exploring the dietary disparities between the Jiangnan area of Zhejiang Province and the historically and culturally related population in Henan Province in central China could prove useful for forecasting the reduction in CVD disease burden through the adoption of the Jiangnan diet in central China. This comparative study has potentially substantial public health significance.

Although previous studies have estimated the contribution of dietary factors to cardiometabolic mortality in China, evidence on long-term changes in diet-attributable CVD mortality burden at the provincial level and on regional contrasts within China remains limited. Using repeated population-based nutrition surveillance data from Zhejiang Province between 2008 and 2024, we examined temporal changes in suboptimal dietary exposures and the corresponding model-based PAFs of CVD mortality. We further compared dietary exposures and estimated PAFs between Zhejiang and Henan Provinces and conducted a counterfactual analysis to quantify the potential reduction in diet-attributable CVD mortality in Henan under the more favorable dietary exposure profile observed in coastal Zhejiang.

## 2. Materials and Methods

### 2.1. Study Design and Sampling Method

This was a repeated cross-sectional study based on multiple rounds of population-based nutrition surveillance conducted in Zhejiang Province between 2008 and 2024, together with comparative survey data from Henan Province in 2015 [23]. Each survey used a multistage stratified cluster sampling design covering urban and rural survey sites in the corresponding province. From 2008 to 2024, a uniform three-stage sampling procedure was implemented. First, several townships (streets) were selected from each survey site. Next, administrative villages (neighborhood committees) were randomly chosen from the selected townships (streets). Finally, households were randomly drawn from the selected villages (committees), and all members of these households were included in the survey. Individuals with major illnesses or other conditions preventing their cooperation were excluded. All survey samples were independent, and efforts were made to avoid repeatedly sampling the same communities across different waves. The study protocol was approved by the Medical Ethics Committee of the National Institute for Nutrition and Health, Chinese Center for Disease Control and Prevention (Approval No. 2022-020).

### 2.2. Study Population

**Figure A1:**
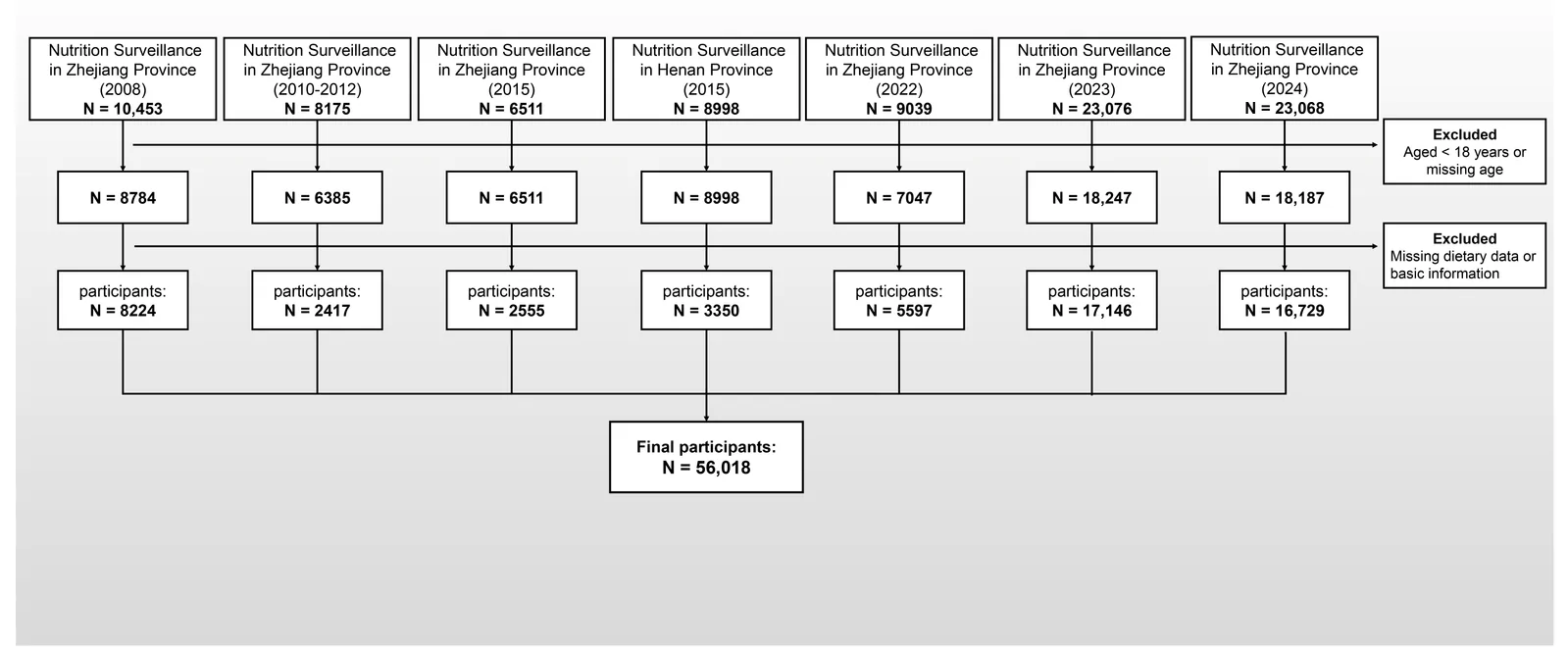
Flow diagram.

Participants met the following inclusion criteria: (1) aged 18 years or over; (2) permanent residency within the sampled communities; and (3) absence of significant diseases or other health issues. A total of 56,018 participants were included. The flowchart illustrating participant inclusion and exclusion is presented in Appendix B Figure A1. Specific details about the survey sites for each year are provided in Table 1.

### 2.3. Data Collection

Investigators were required to hold a degree in medicine or public health. Those who had received unified specialized training, assessment, and certification were assigned to collect information through questionnaires and a dietary survey. A questionnaire survey was used to collect basic and health-related data regarding the age, educational level, occupation, and diagnostic status of CVDs and other chronic diseases. Dietary assessment was based on a combination of three days of consecutive 24-h recalls (3 d–24 h) at the individual level and the weights of oils and seasonings used at the household level taken over the same three-day period. Individual dietary intake data were collected by asking every household member to report all food they had consumed at home and outside the home over the previous 24 h. The three consecutive days included two working days and one rest day. Household oil and seasoning consumption was determined by a weighing and measuring technique that examined changes in the inventory from the beginning to the end of the survey. Then, based on the household cooking schedule and the energy intake of each family member, the consumption of oil and seasonings was allocated to each individual. If guests were present in the household, their shares were also accounted for in the allocation.

### 2.4. Assessment of Dietary Intake

The food intake of residents collected through dietary surveys was classified and aggregated into food groups based on food codes in the Food Composition Table [24], followed by standardization using the standard person coefficient [25]. Rice and its products, as well as wheat and its products, were converted according to the energy densities of rice and wheat, respectively. Miscellaneous beans were converted based on the protein content of adzuki beans, while soybeans and their products were converted using the protein content of dried soybeans. The condiment salt was converted based on the sodium content in the salt and sauce categories. Dark-coloured vegetables were defined as vegetables containing ≥500 μg of carotene per 100 g of edible portion. The proportion of dark-coloured vegetables was calculated relative to total vegetable intake and was used only for descriptive regional comparisons rather than as an exposure variable in the PAF model.

The food composition database used for each survey wave corresponded to the most current Chinese Food Composition Table available during that survey period, in order to reflect updated information on nutrient composition and improve the accuracy of dietary nutrient estimation. The 2000 Chinese Dietary Reference Intakes [26] and the 2002 Food Composition Table [24] were applied to the dietary data analysis in 2008. For the dietary survey data from 2010 to 2015, the 2013 Chinese Dietary Reference Intakes [25] and the 2009 Food Composition Table [27] were used. For the dietary data analysis from 2022 to 2024, the 2013/2023 Chinese Dietary Reference Intakes [28] and the 2019 Food Composition Table [29] were adopted.

The standard person coefficient reflects the relative energy requirement of each household member compared with a reference adult. Household members were converted into standard-person equivalents using these coefficients, and household food and condiment consumption was divided by the total number of standard-person equivalents to obtain intake per standard person.

Dietary exposures were dichotomized according to Dietary Guidelines for Chinese Residents and recommended or optimal intake thresholds of previous comparative risk-assessment studies to estimate the prevalence of suboptimal intake and corresponding threshold-based PAFs. This approach was selected for interpretability and consistency with public-health dietary recommendations, although it does not capture the full continuous dose–response relationship. The final definitions of suboptimal levels of dietary factors were as follows: daily intake of condiment salt ≥ 5 g/standard person; red meat ≥ 100 g/standard person; processed meat ≥ 50 g/standard person; fruits < 300 g/standard person; vegetables < 300 g/standard person; aquatic products < 50 g/standard person; nuts < 20 g/standard person; whole grains < 35 g/standard person; sugar-sweetened beverages (SSBs) ≥ 50 g/standard person and omega-3 fatty acids (ω-3FAs) < 0.175 g/standard person. Because the largest discrepancy between the survey sample distribution and the corresponding provincial population structure was observed for urban–rural residence, standardization was performed according to provincial urban–rural population proportions [30,31]. Age- and sex-specific estimates were additionally examined in stratified analyses.

### 2.5. Quality Control

To ensure the comparability of samples across successive surveillance cycles, the following core measures were implemented: unified and standardized survey tools (including standardized dietary questionnaires) were adopted for data collection to guarantee the comparability of data across all surveillance waves; the survey process, interview scripts, and detailed information recording rules were clearly defined to avoid data bias caused by heterogeneity in survey tools; and a unified food classification and coding standard was established, whereby all foods were systematically categorized into groups (e.g., grains and tubers, vegetables and fruits, livestock and poultry meats, fish and shrimp, dairy and eggs, and soy products) to ensure a high degree of consistency in the statistical standards for food intake across different surveillance cycles. Furthermore, all investigators had undergone unified specialized training and assessment certification, and three-level training (national–provincial–county or provincial–municipal–county) was conducted prior to the initiation of surveys to ensure proficiency in survey technical specifications, food classification and coding rules, and data entry standards and to minimize systematic errors caused by human operation. During the survey, a three-level on-site supervision mechanism and a data verification mechanism were established, and data completeness and accuracy were guaranteed through logical validation, removal of duplicate data, and identification of outliers to lay a solid data foundation for the comparative analysis of samples across cycles.

Because the urban–rural composition of the survey samples differed from that of the corresponding provincial population, dietary intake and the prevalence of suboptimal dietary exposures were standardized according to the provincial urban–rural population distribution. The standardized mean intake was calculated as:

$${\bar{X}}_{std}=\sum_{i}W_{i}X_{i}$$

and the standardized prevalence was calculated as:

$$P_{std}=\sum_{i}W_{i}P_{i}$$

where ${\bar{X}}_{std}$ is the urban–rural standardized mean; ${\bar{X}}_{i}$ is the stratified mean; $W_{i}$ is the weight of the urban–rural population proportion; $P_{std}$ is the urban–rural standardized suboptimal dietary factor prevalence; and $P_{i}$ is the stratified suboptimal dietary factor prevalence.

### 2.6. Data Sources of Relative Risks

Relative-risk estimates for the associations between dietary factors and cardiovascular outcomes were obtained from published systematic reviews and meta-analyses of prospective cohort studies. Evidence from Asian populations was prioritized when available. When multiple eligible meta-analyses were identified, preference was given to more recent studies with larger sample sizes, prospective study designs, clearly defined dietary exposures, adjustment for major cardiovascular risk factors, and effect estimates that could be aligned with the exposure definitions used in the present study.

For dietary factors for which Asian-specific evidence was unavailable, pooled estimates from high-quality meta-analyses conducted in other populations were used. The selected effect estimates were harmonized with the predefined suboptimal-intake thresholds used in the PAF model. For each dietary factor, the selected RR, 95% CI, exposure definition, outcome, source population, and reference are provided in Appendix A Table A1 [32,33,34,35,36,37,38,39].

### 2.7. Statistical Analyses

Descriptive statistics were computed as follows: continuous variables with normal distributions were summarized using mean ± standard deviation (SD). One-way analysis of variance was employed to compare differences in the intake of various foods among populations of different age groups in the same surveillance cycle, while an independent samples *t*-test was used to examine differences in food intake between different regions as well as between genders during the same surveillance cycle. Sampling weights and design-based variance estimation incorporating strata and primary sampling units were not applied in the present analysis. Accordingly, the estimates should be interpreted as standardized model-based estimates rather than fully survey-weighted population estimates. All statistical tests were two-tailed, with the significance level set at α = 0.05. A *p* value < 0.05 was considered statistically significant.

Assuming a causal relationship between each dietary factor and CVD mortality, we computed the PAF to determine the proportional reduction in deaths achievable if each risk factor was reduced to its theoretical minimum-risk exposure level [40]. To quantify the preventable burden of CVD mortality, we employed a population-level comparative risk assessment framework [41]. Specifically, we estimated the number and proportion of CVD deaths that could have been averted under the following scenario: current exposures to specific dietary risk factors are shifted to a counterfactual optimal distribution, with all other risk factors held constant. We assessed the independent and combined effects of suboptimal intakes of 10 dietary factors. For this purpose, participants were stratified into 24 subgroups by sex, age group (18–45, 45–60, and ≥60 years), urban–rural status, and coastal regions. The calculation formula is as follows:

$$PAF\;=\;\frac{p_{e}\;({RR}_{e}\;-\;1)}{p_{e}\;\left({RR}_{e}\;-\;1\right)\;+\;1}$$

$p_{e}$: Proportion of the total population that is exposed;
${RR}_{e}$: Relative risk of the exposed group compared with the unexposed group.

$${PAF}_{combined}=1-\prod_{i=1}^{n}\left(1-{PAF}_{i}\right)$$

${PAF}_{combined}$: Total population-attributable fraction for multiple co-acting exposure factors;
*n*: Number of exposure factors considered;
${PAF}_{i}$: Population-attributable fraction for the ith individual exposure factor.

The method for estimating the combined PAF was based on established comparative risk assessment approaches and was consistent with that used in a previous nationally representative study of diet-attributable CVD mortality in China [12,41]. To minimize obvious overlapping attribution, dietary factors representing closely nested or redundant exposures were not simultaneously included. Nevertheless, because correlations among dietary exposures cannot be fully eliminated, the combined PAF should be interpreted as a model-based estimate under the assumptions of the comparative risk assessment framework. To quantify uncertainty in the PAF estimates arising from the published relative-risk estimates, we performed Monte Carlo simulations. For each dietary factor, the natural logarithm of the RR was assumed to follow a normal distribution, with its standard error derived from the published 95% CI as [ln(RR upper) − ln(RR lower)]/(2 × 1.96). In the Monte Carlo simulation, dietary-exposure prevalence was held fixed, and only uncertainty in the published RR estimates was propagated. Therefore, the resulting 95% uncertainty intervals reflect uncertainty in the published RRs only and do not represent the total uncertainty of the PAF estimates. In each simulation, one RR was sampled for each dietary factor and applied consistently across all population strata. Factor-specific and combined PAFs were then recalculated, while the observed prevalence of suboptimal dietary exposure was held fixed. The 2.5th and 97.5th percentiles of the simulated PAF distributions were reported as RR-based 95% uncertainty intervals (UIs).

All statistical analyses were performed using SAS software (Version 9.4).

## 3. Results

### 3.1. Basic Information

A total of 52,668 participants were included in the six Zhejiang nutrition surveillance waves conducted between 2008 and 2024. An additional 3350 participants from Henan Province in 2015 were included in the cross-provincial comparison. The historical data from some years lacked basic demographic information; therefore, the samples with complete baseline characteristics were assessed, and they indicated the following: males accounted for 47.5% and females for 52.5%; 34.4% of the participants were aged 18–44 years, 27.1% were aged 45–59 years, and 38.5% were aged ≥ 60 years; 43.5% were urban residents and 56.5% were rural residents (Table 2). Overall, the sample population was well balanced in terms of gender, age, and urban–rural distribution.

### 3.2. Distribution and Temporal Changes in Suboptimal Dietary Factors

**Figure 2:**
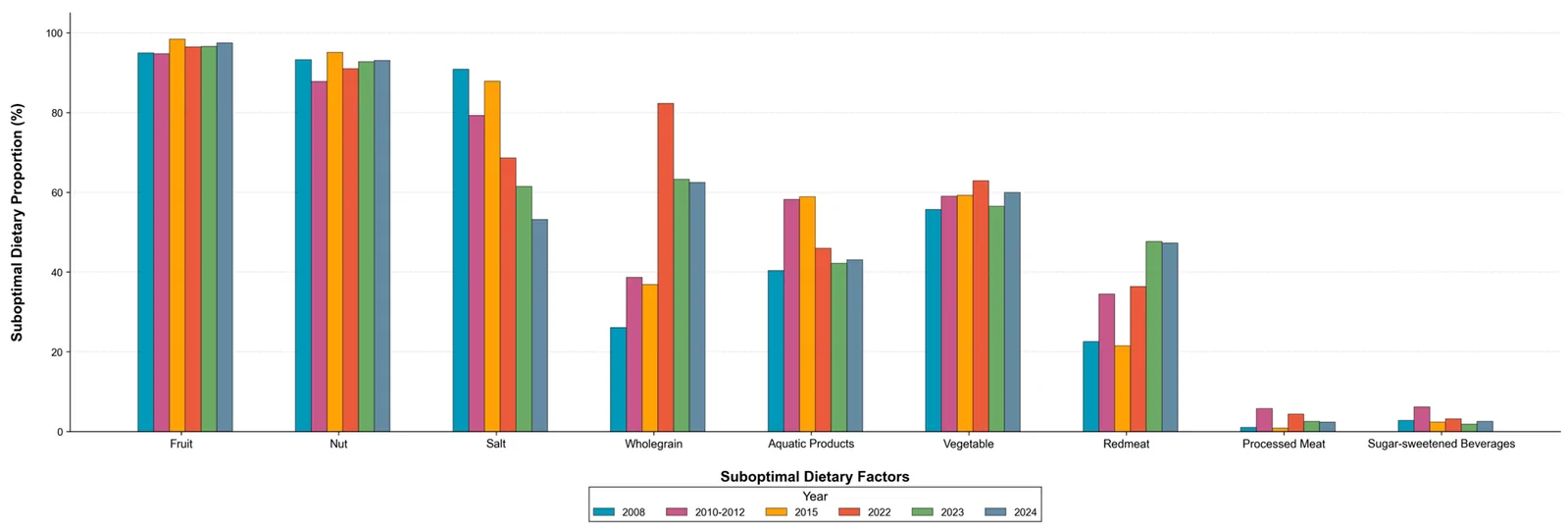
Prevalence of selected suboptimal dietary factors in Zhejiang Province, 2008–2024. Figure legend: Seven of the 10 dietary factors included in the comparative risk-assessment model are shown for visual clarity; all 10 dietary factors were included in the PAF calculations.

Among the 10 dietary risk factors assessed in this serial population-based survey, relatively low rates of inadequate consumption were noted for aquatic products, whole grains, and vegetables, coupled with a notable reduction in excessive salt intake, in alignment with the typical characteristics of the Jiangnan diet. Figure 2 presents seven selected suboptimal dietary factors for visual clarity; all 10 dietary factors were included in the subsequent PAF analyses. In contrast, insufficient fruit and nut intake was evident in almost 90% of the study population. Moreover, excessive red meat consumption steadily increased (Figure 2). The urban–rural disparities in most suboptimal dietary factors narrowed over the 16-year period, although notable differences by sex and age persisted (Table 3). Specifically, the burden of suboptimal dietary factors was consistently higher among young adults and males than among older adults and females. Geographically, the prevalence of insufficient aquatic product consumption was lower in coastal areas than in noncoastal areas. Overall, some improvement was noted in the population-level prevalence of suboptimal dietary factors during 2008–2024.

### 3.3. Population-Attributable Proportion of Suboptimal Dietary Factors for Cardiovascular Diseases

**Figure 3:**
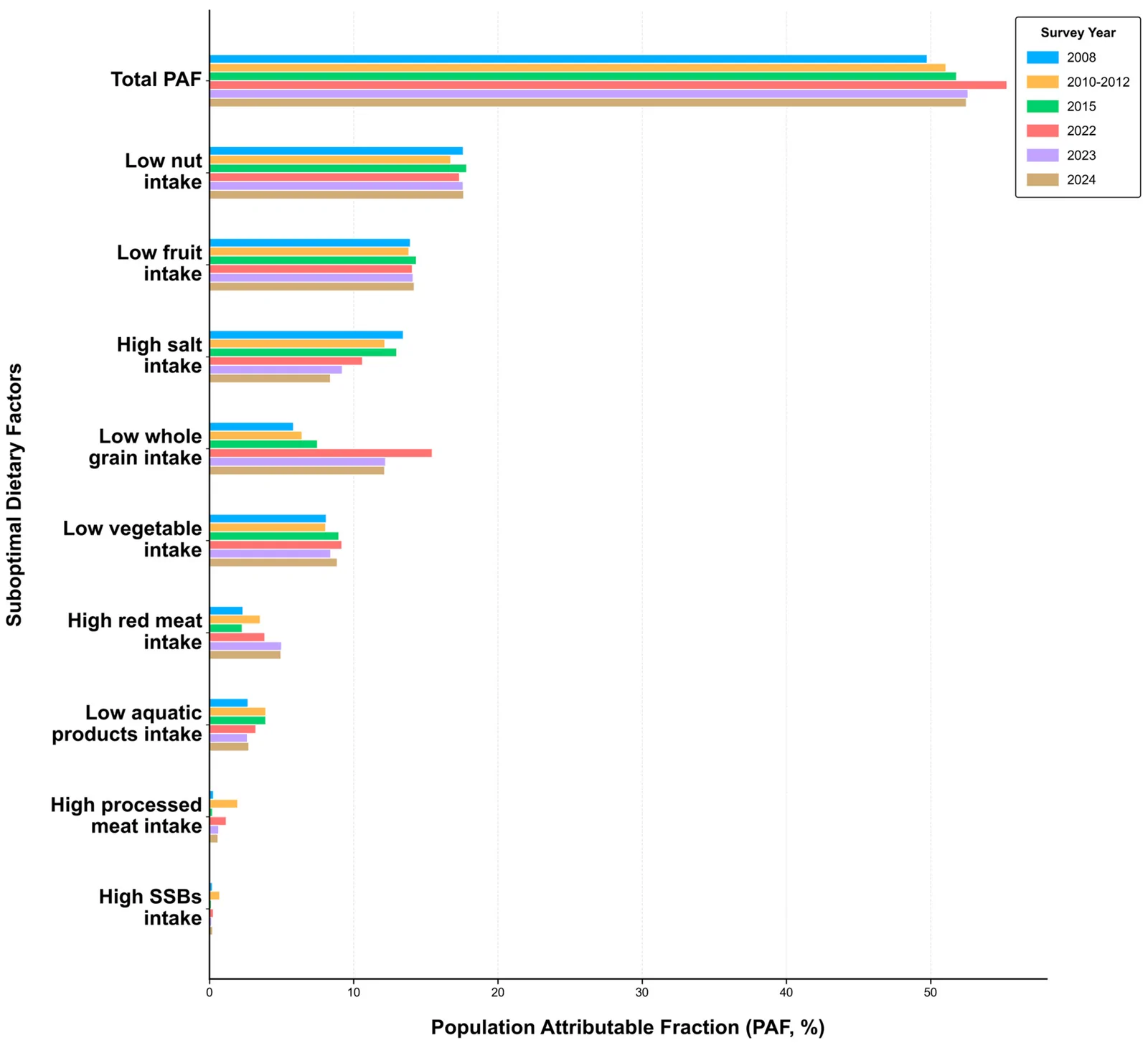
Population attributable fractions of CVD mortality associated with suboptimal dietary factors in Zhejiang Province, 2008–2024.

**Figure 4:**
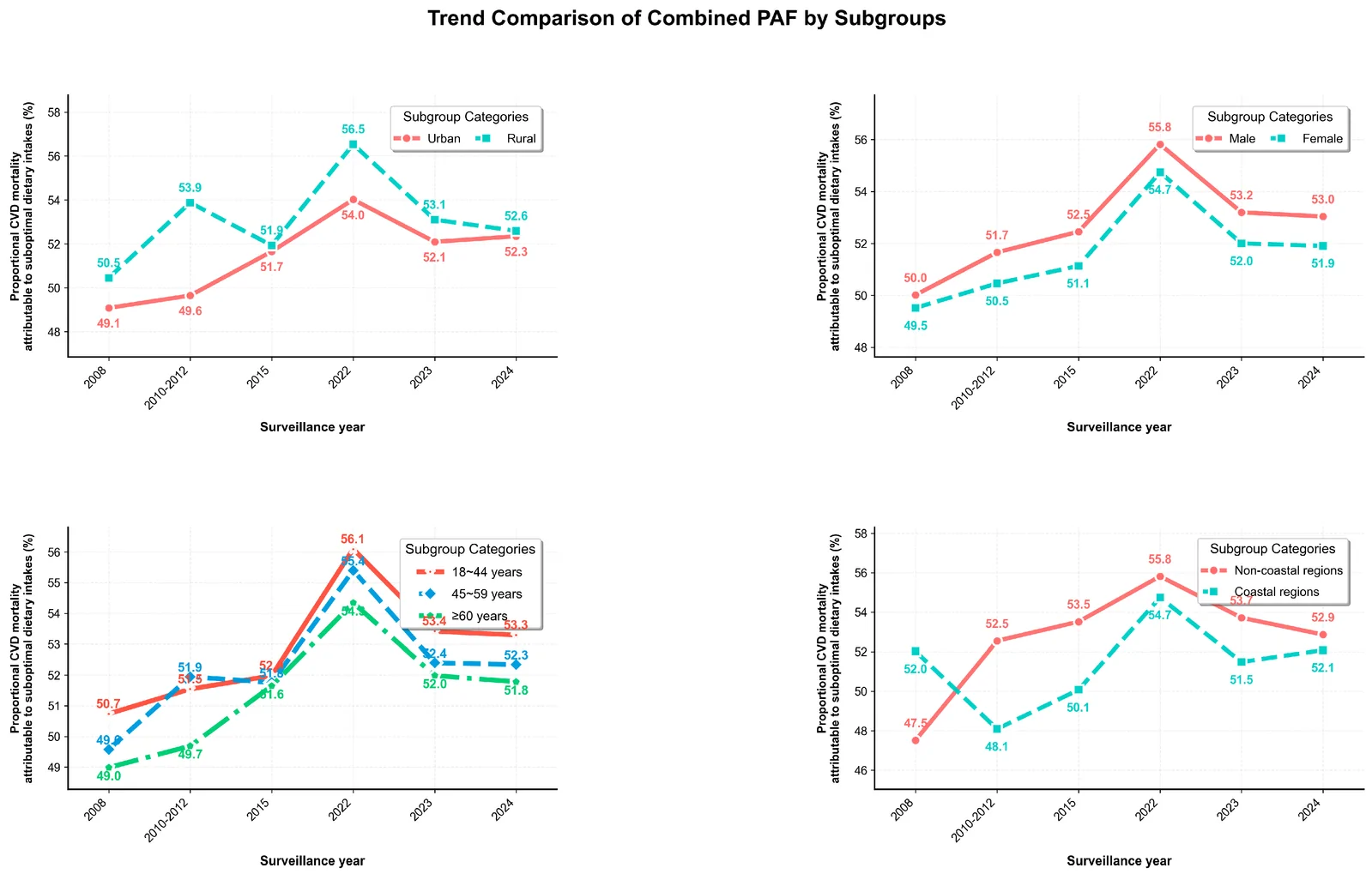
Subgroup analysis of proportional CVD mortality attributable to suboptimal dietary intakes.

The estimated suboptimal diet-attributable CVD mortality burden remained relatively stable at approximately 50–55% across 2008 to 2024 (Figure 3). This was primarily reflected in the low intake of several protective foods (e.g., insufficient consumption of nuts, fruit, whole grains, and vegetables) and among specific populations (i.e., rural residents, males, young adults, and residents of noncoastal regions) (Figure 4). The estimated diet-attributable CVD mortality burden based on comparative risk assessment was 49.8% (95% UI, 40.8–57.4) in 2008, increased numerically to 55.3% (48.1–61.6) in 2022, and was 52.5% (45.6–58.6) in 2024. The reported 95% uncertainty intervals reflect uncertainty in the published RR estimates only, as dietary-exposure prevalence was held fixed in the Monte Carlo simulation. The total dietary-attributable burden remained substantial and showed a slight upward trend over the 16-year span. Among all the suboptimal dietary factors, insufficient intake of nuts, fruit, and whole grains ranked among the top three factors in terms of the PAF. In contrast, the excessive intake of processed meat and sugar-sweetened beverages had the lowest PAF rankings. The PAF for excessive condiment salt intake fell numerically from 13.5% (95% UI 1.8–23.7) to 8.3% (1.1–15.5). The prevalence of excessive salt intake decreased over time, *p*_trend_ < 0.001. In contrast, the PAF for excessive red meat consumption more than doubled, rising numerically from 2.3% (1.2–3.5) to 5.0% (2.6–7.3). The prevalence of excessive red meat consumption increased over time, *p*_trend_ < 0.001.

**Figure A2:**
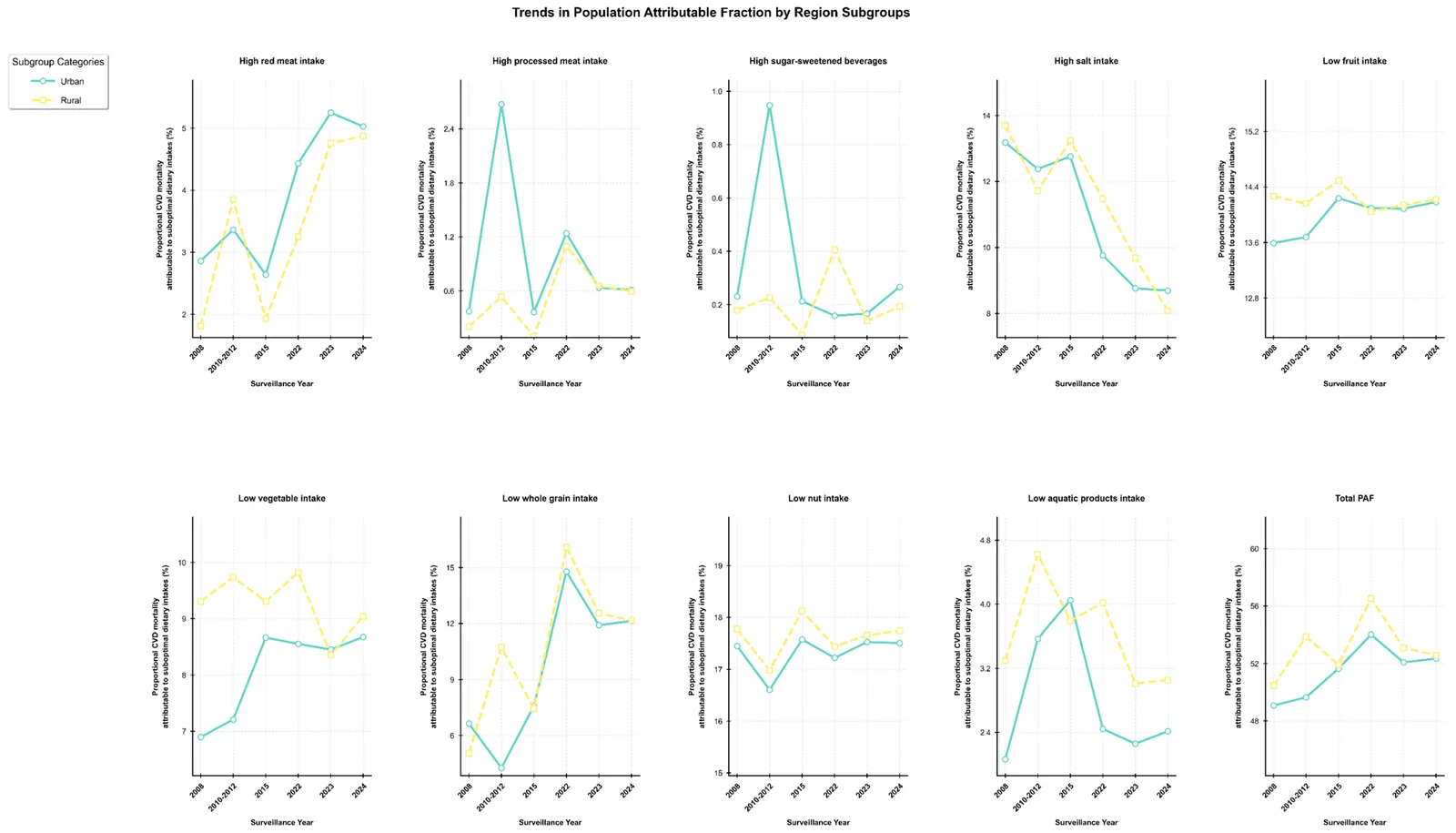
Region-Specific Subgroup Analysis of Proportional CVD mortality Attributable to Suboptimal Dietary Intakes.

**Figure A3:**
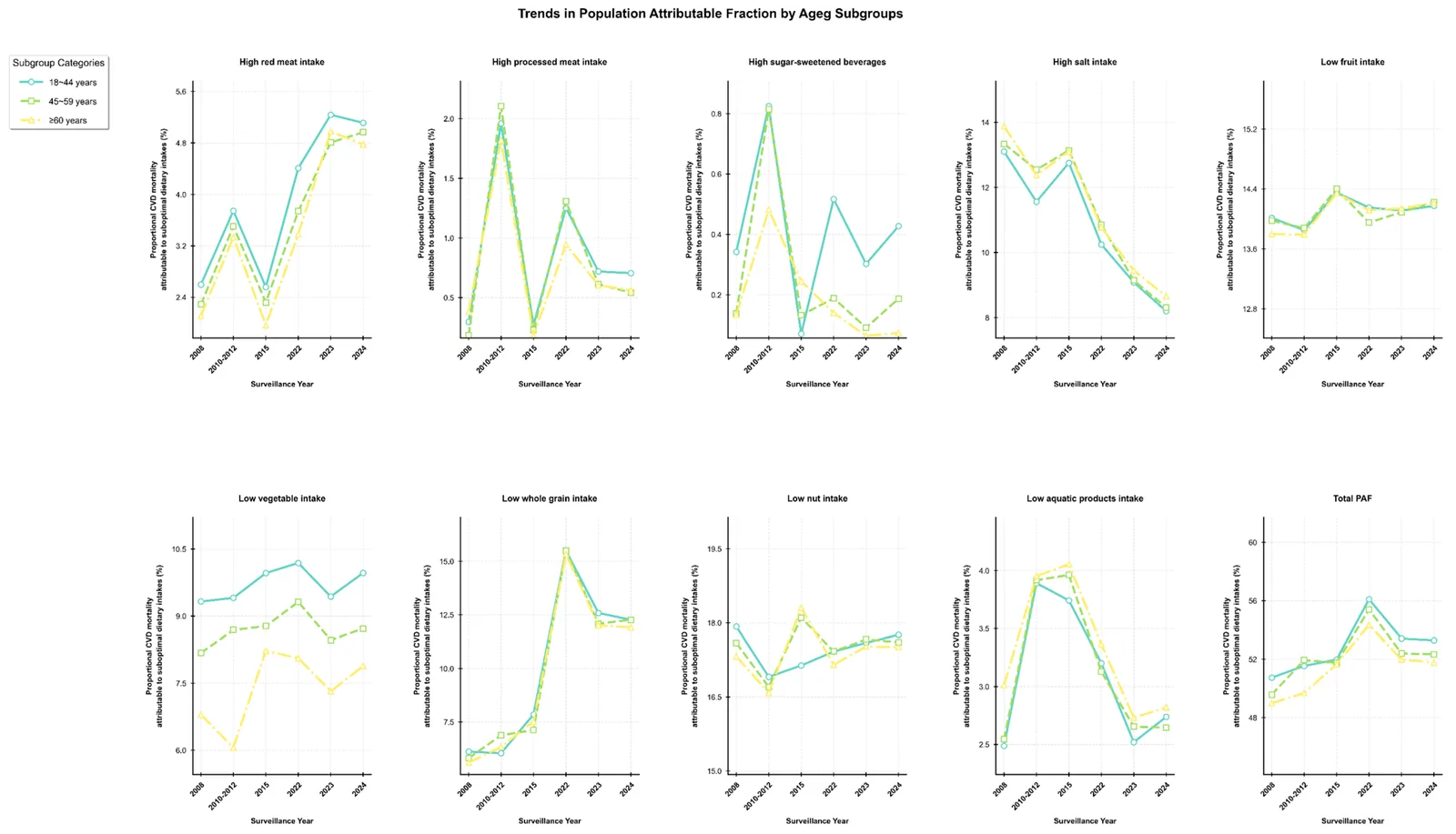
Age-Specific Subgroup Analysis of Proportional CVD mortality Attributable to Suboptimal Dietary Intakes.

**Figure A4:**
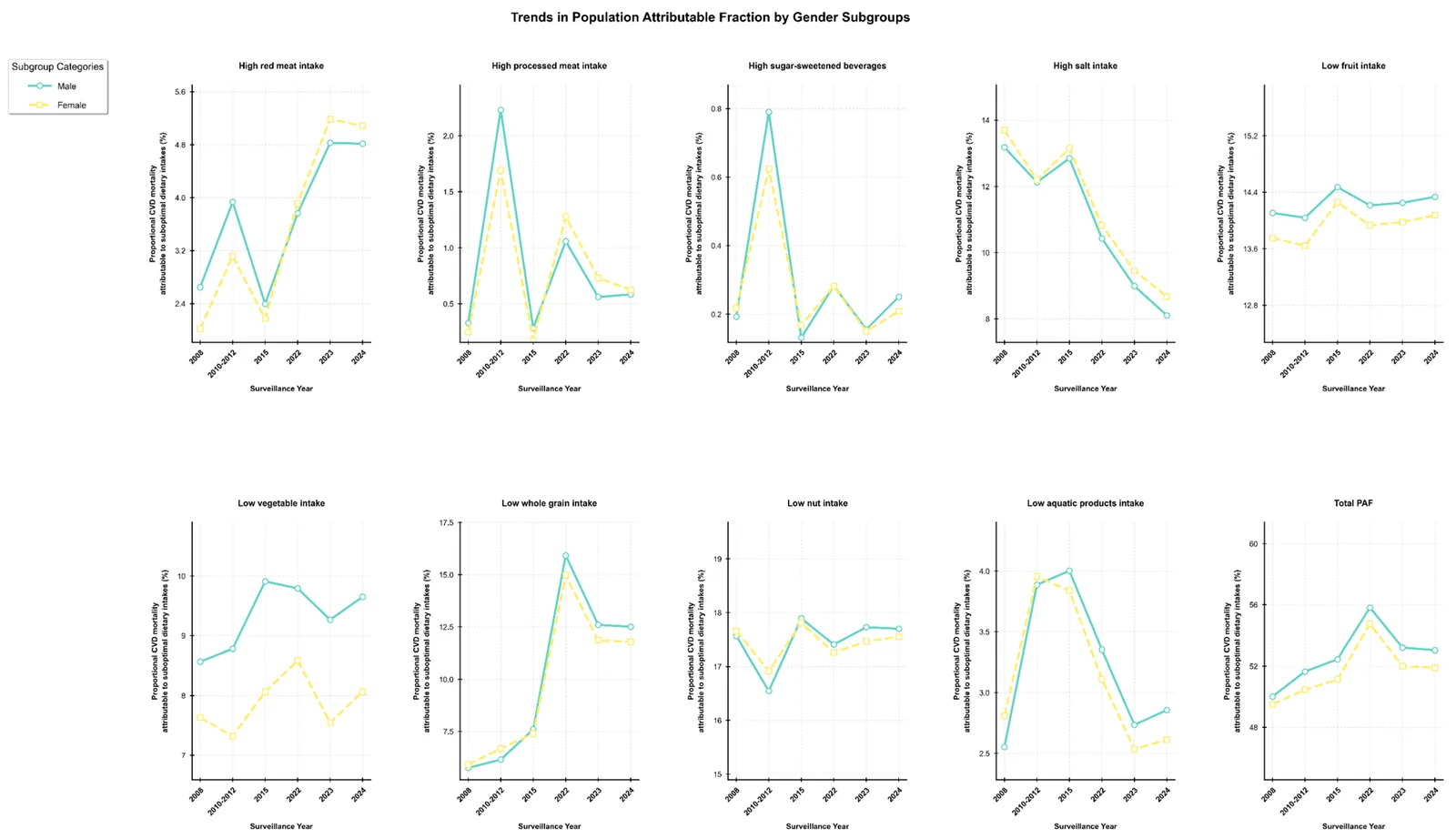
Sex-Specific Subgroup Analysis of Proportional CVD mortality Attributable to Suboptimal Dietary Intakes.

**Figure A5:**
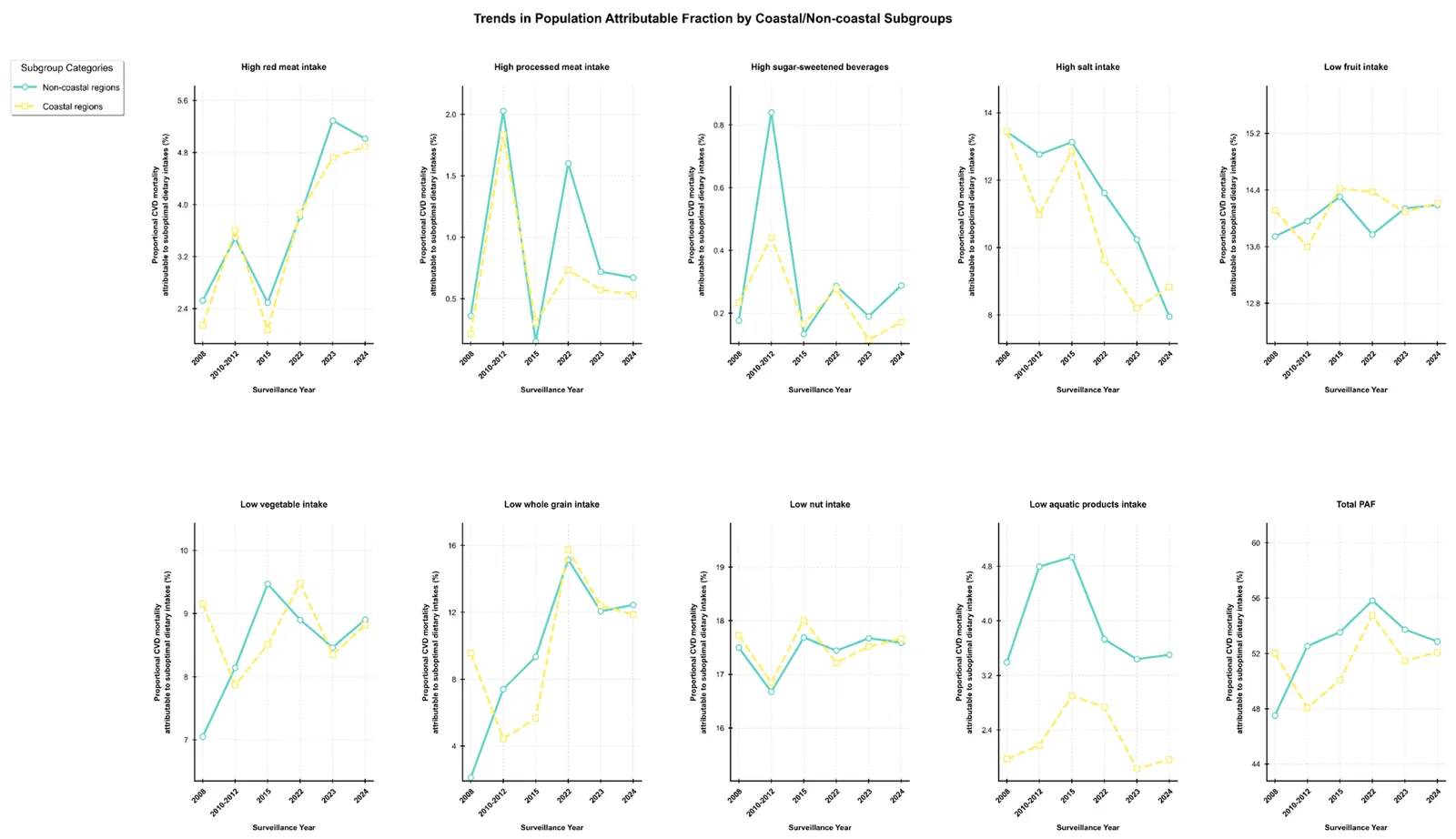
Coastal-Specific Subgroup Analysis of Proportional CVD mortality Attributable to Suboptimal Dietary Intakes.

Subgroup analysis results (Figure 4 and Appendix B Figure A2, Figure A3, Figure A4 and Figure A5) revealed a higher combined dietary risk burden among rural residents, males, younger adults, and people living in noncoastal areas. The combined dietary PAF in coastal Zhejiang was lowest in 2010–2012, at 48.1% (95% UI, 40.1–55.2). The urban–rural disparity was particularly evident for insufficient vegetable and aquatic product intake, while the gender and age group gap was largely driven by the low consumption of vegetables.

### 3.4. Comparison of Zhejiang and Henan Provinces and Prediction of Potentially Preventable Deaths

**Figure 5:**
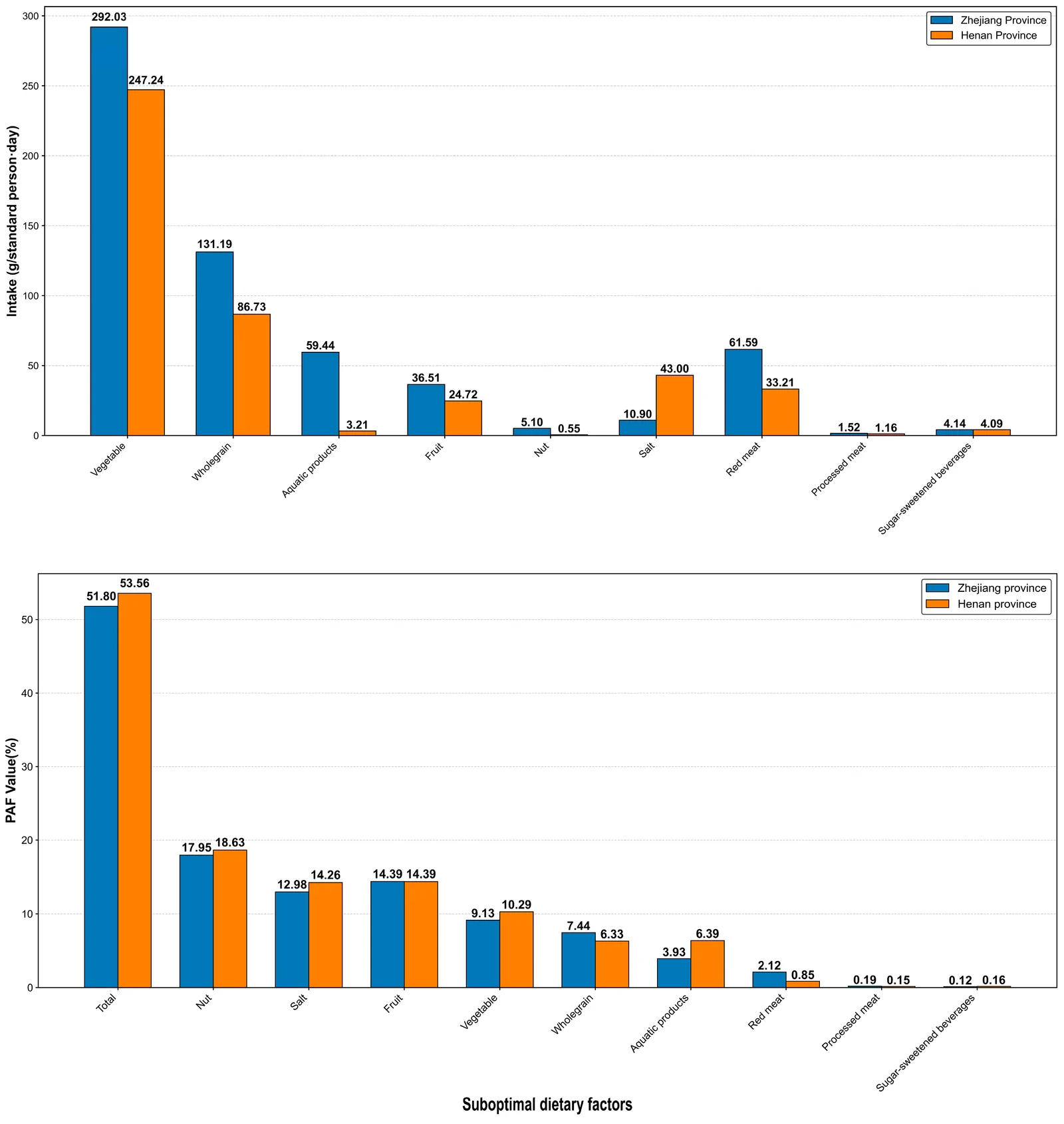
Comparison of dietary intake and PAF of suboptimal dietary factors between Zhejiang Province and Henan Province.

The intakes of vegetables, whole grains, aquatic products, fruits, and nuts were higher in Zhejiang Province than in Henan Province, with Zhejiang’s aquatic product intake nearly 20 times that of Henan. The intake of dark-colored vegetable proportion (32.0%) was higher in Zhejiang than in Henan (21.5%) (Figure 5), while that of condiment salt was lower in Zhejiang compared to Henan. Although red meat intake was higher in Zhejiang Province, the proportion of red meat in animal-derived foods was significantly higher in Henan (84.6%) than in Zhejiang (51.5%). These findings are consistent with the typical characteristics of the Jiangnan diet. Moreover, a comparison of the PAF of diet-related CVD mortality between Zhejiang and Henan Provinces revealed a lower proportion of CVD mortality attributable to suboptimal diets in Zhejiang than in Henan, with the disparity primarily stemming from insufficient intake of CVD-protective foods, such as aquatic products, omega-3 fatty acids, nuts, and vegetables.

Based on these research findings, the PAF of CVD mortality attributable to suboptimal diets was relatively lower in Zhejiang Province, especially among females living in coastal areas. To quantify the public health value of this healthy dietary pattern, further estimates were conducted to establish the theoretical number of CVD deaths that could be prevented if Henan were to adopt the dietary patterns of coastal areas in Zhejiang. According to the 2016 Statistical Yearbook, the permanent population in Henan Province was 94.8 million. The PAF of CVD mortality related to suboptimal diets was 54.83% in Henan Province, whereas it was only 48.0% in Zhejiang Province, resulting in a PAF difference of 6.83%. The CVD mortality rate adopted in this study was 364.1 per 100,000 population [42]. The number of preventable deaths was calculated using the following formula (dividing by 100,000 to convert the mortality rate from “per 100,000 population” to the total population scale):

Preventable deaths = Population size of Henan Province × CVD mortality rate × PAF difference ÷ 100,000. The results indicate that approximately 23,575 annual CVD deaths could potentially be averted in Henan under the specified counterfactual assumptions, should it adopt a dietary pattern similar to that of Zhejiang Province. This value represents a model-based point estimate; a complete uncertainty interval could not be derived because the variance–covariance structure required to jointly propagate uncertainty in the exposure prevalences and correlated dietary factors was unavailable.

## 4. Discussion

This study used repeated nutritional surveillance data spanning 16 years (2008–2024) to characterize temporal changes in the burden of dietary factor–attributable CVD in Zhejiang Province. Low rates of inadequate consumption of aquatic products, whole grains, and vegetables, together with a marked reduction in excessive salt intake, are hallmarks of the Jiangnan dietary pattern, as revealed here. When compared with the diet consumed in Henan Province, the dietary intake profile was notably healthier in Zhejiang; however, even with this favorable pattern, suboptimal dietary factors remained major risk factors for CVD mortality in Zhejiang over the 16-year period. The lowest dietary PAF for CVD mortality was observed in the coastal female population of Zhejiang, and our calculations indicated that scaling up this healthier coastal dietary pattern could avert approximately 23,575 annual CVD deaths in Henan Province under the specified counterfactual assumptions.

Some studies have reported that CVDs are attributable to physiological factors, such as BMI (PAF: 18%), abdominal obesity (PAF: 9.58%), hypertension (PAF: 14.04–15.1%), and behavioral factors (PAF: 10.68%, primarily smoking) [43,44,45]. Suboptimal dietary factors represent an important modifiable contributor to the CVD burden. A previous population-based cross-sectional study revealed that the PAF of cardiometabolic mortality (including cardiovascular disease and type 2 diabetes) associated with a suboptimal diet was 62.2% in 1982, 57.9% in 1992, 56.2% in 2002, and 51.0% in 2010–2012, indicating a gradual yet limited decline over three decades [12]. However, direct comparison with our data is not feasible due to differences in the definition of suboptimal dietary factors and diseases, as well as the survey years. We compared the average daily intake of various food groups per standard person in Zhejiang Province with the national average determined from 2010–2012 and found that Zhejiang’s intake exceeded the national level for vegetables (299.3 vs. 248 g/person/day), fruits (61.8 vs. 39.3 g/person/day), red meat (89.8 vs. 64.4 g/person/day), aquatic products (57.0 vs. 23.0 g/person/day), eggs (25.6 vs. 23.5 g/person/day), milk and dairy products (26.2 vs. 23.1 g/person/day), and nuts (7.4 vs. 3.4 g/person/day). In contrast, cooking salt intake was lower in Zhejiang (10.8 vs. 13.2 g/person/day). Overall, the average food intake in Zhejiang was closer than the national average in terms of the Dietary Pagoda’s recommended range [14]. Subgroup analyses showed lower estimated dietary PAFs among females, urban residents, older adults, and coastal populations. These differences may be related to variations in dietary behaviors, nutrition-related knowledge, and access to cardioprotective foods. However, these factors were not directly measured in the present study and should therefore be regarded as possible explanations rather than confirmed mechanisms.

After the Southern Song Dynasty moved its capital to Hangzhou, large-scale migration from Henan reshaped the city’s demographic structure, giving the two populations shared historical and dietary traditions that support the comparability of their dietary patterns. Our comparison of the dietary intakes between the Henan and Zhejiang populations revealed that, with the exception of red meat intake, the mean dietary intake levels across the other food categories were closer to the recommended dietary guidelines in Zhejiang than in Henan. Compared with the Henan residents, the residents in Zhejiang Province consumed a diet with a lower proportion of red meat and a higher proportion of dark-colored vegetables. Current research indicates that replacing red meat with white meat can effectively reduce the CVD mortality rate [46], while the abundant carotene in dark-colored vegetables has an antioxidant effect and serves as an important protective factor against CVDs. However, the prevalent finding of suboptimal dietary patterns and PAFs for cardiovascular mortality suggested a narrow gap between the two provinces, implying a relatively high proportion of undesirable dietary patterns in Zhejiang. The findings also highlight that a subgroup in Henan Province has a high prevalence of extreme suboptimal dietary intake and merits targeted attention. Within Zhejiang, we also observed a lower prevalence of suboptimal diets among coastal cities. Our calculations indicated that, by scaling up this coastal dietary pattern, approximately 23,575 CVD deaths per year were estimated to be potentially avertable in Henan Province under the specified counterfactual assumptions.

Some features of the Jiangnan dietary pattern, such as a relatively high consumption of vegetables and aquatic products, may resemble components of other cardioprotective dietary patterns, including the Mediterranean diet. However, the two dietary patterns differ substantially in food sources, cooking practices, and cultural context, and direct equivalence should not be inferred.

This study extends previous national comparative risk assessments by providing a 16-year provincial perspective on dietary changes and by applying a cross-provincial counterfactual framework to examine regional differences in diet-attributable CVD mortality.

Several limitations should be acknowledged. First, the repeated cross-sectional design precludes individual-level causal inference, and residual differences in population composition across survey waves may have influenced temporal comparisons despite urban–rural standardization. Second, dietary intake assessed by 3-day 24-h recalls and household condiment weighing may be subject to recall and measurement error, and the use of different food composition tables across periods may introduce some methodological heterogeneity. Third, dichotomizing dietary exposures according to predefined thresholds may not fully capture dose–response relationships. Fourth, the PAF calculations relied on relative-risk estimates from published meta-analyses rather than local prospective cohorts, which may limit their transportability; correlations among dietary factors may also affect the combined PAF. Finally, the estimated PAFs and potentially avertable CVD deaths are model-based counterfactual estimates and should not be interpreted as the effects that would necessarily be achieved through an actual dietary intervention.

## 5. Conclusions

Despite having a healthier dietary intake profile than central China’s regions (e.g., Henan Province), the suboptimal factors still contributed to a high burden for CVD mortality in Zhejiang Province, with no significant improvement observed over the past 16 years. Key concerns include an insufficient intake of nuts, fruits, and whole grains, particularly among rural residents, young and middle-aged adults, males, and populations in noncoastal areas. Future research will focus on refining the distinctive Jiangnan diet, with the aim of promoting its adoption to inform evidence-based policies for reducing the CVD burden, especially in regions with a heavy burden of CVD.

## Figures and Tables

**Table 1:** Sample inclusion in nutrition surveillance surveys, 2008–2024.

Year	Number of Survey Sites (*n*)	Number of Urban Survey Sites (*n*)	Number of Rural Survey Sites (*n*)	Number of Respondents (*n*)
2008	22	11	11	8224
2010–2012	6	3	3	2417
2015 (Zhejiang)	10	4	6	2555
2015 (Henan)	14	8	6	3350
2022	16	8	8	5597
2023	90	37	53	17,146
2024	90	37	53	16,729
Total	234	100	134	56,018

**Table 2:** Baseline Characteristics of the Survey Population [*n* (%)].

Year	Gender		Age Group, Year			Region	
	Male	Female	18~44	45~59	≥60	Urban	Rural
2008	3925 (47.7%)	4299 (52.3%)	3646 (44.3%)	2710 (33.0%)	1868 (22.7%)	4238 (51.5%)	3986 (48.5%)
2010–2012	1159 (48.0%)	1258 (52%)	740 (30.6%)	881 (36.5%)	796 (32.9%)	1143 (47.3%)	1274 (52.7%)
2015 (Zhejiang)	1238 (48.5%)	1317 (51.5%)	555 (21.7%)	885 (34.6%)	1115 (43.6%)	500 (19.6%)	2055 (80.4%)
2015 (Henan)	1565 (46.7%)	1785 (53.3%)	841 (25.1%)	1198 (35.8%)	1311 (39.1%)	2130 (63.6%)	1220 (36.4%)
2022	2561 (45.8%)	3036 (54.2%)	1894 (33.8%)	1805 (32.2%)	1898 (33.9%)	3034 (54.2%)	2563 (45.8%)
2023	8256 (48.2%)	8890 (51.8%)	5802 (33.8%)	3975 (23.2%)	7369 (43%)	7098 (41.4%)	10,048 (58.6%)
2024	7899 (47.2%)	8830 (52.8%)	5503 (32.9%)	4015 (24%)	7211 (43.1%)	6886 (41.2%)	9843 (58.8%)
Total (Zhejiang)	25,038 (47.5%)	27,630 (52.5%)	18,140 (34.4%)	14,271 (27.1%)	20,257 (38.5%)	22,899 (43.5%)	29,769 (56.5%)
Total (including Henan)	26,603 (47.5%)	29,415 (52.5%)	18,981 (33.9%)	15,469 (27.6%)	21,568 (38.5%)	25,029 (44.7%)	30,989 (55.3%)

**Table 3:** Prevalence of population with main suboptimal dietary factors and subgroup comparisons (2008–2024).

Year			*N*	Salt	Fruit	Vegetable	Aquatic Products	Nut	Wholegrain	Red Meat
2008	Total		8224	90.9	95.0	55.7	62.2	93.3	55.7	22.6
	Region	Urban	4238	**88.7**	**92.6**	**47.8**	**53.9**	**92.7**	**47.8**	**27.5**
		Rural	3986	**93.2**	**97.6**	**64.1**	**71.0**	**93.9**	**64.1**	**17.4**
	Age, year	18~44	3646	**88.9**	**95.5**	**63.1**	**61.6**	**95.0**	**63.1**	**24.6**
		45~59	2710	**90.8**	**95.2**	**54.1**	**60.0**	**92.7**	**54.1**	**21.6**
		≥60	1868	**94.9**	**94.0**	**43.8**	**66.5**	**90.8**	**43.8**	**20.1**
	Gender	Female	4299	**92.8**	**93.5**	**52.0**	62.8	93.5	**59.8**	**20.0**
		Male	3925	**88.8**	**96.7**	**59.8**	61.6	93.0	**52**	**25.4**
	Coastal regions	No	4847	91.0	**94.0**	**50.0**	**73.4**	**92.8**	50	**23.6**
		Yes	3377	90.7	**96.6**	**63.9**	**46.0**	**93.9**	63.9	**21.1**
2010–2012	Total	Total	2417	79.3	94.8	59.0	80.2	87.8	**48.7**	34.5
	Region	Urban	1274	78.4	**97.0**	**68.3**	**90.0**	88.9	**68.3**	**36.6**
		Rural	1143	80.3	**92.4**	**48.7**	**69.2**	86.7	69.3	**32.3**
	Age, year	18~44	881	**82.4**	95.6	**64.2**	**81.0**	**87.6**	64.2	34.6
		45~59	796	**79.3**	93.8	**43.7**	**75.8**	**85.9**	43.7	32.9
		≥60	740	**75.7**	95.0	**69.3**	**83.9**	**90.1**	**63.4**	36.2
	Gender	Female	1258	78.9	**93.0**	**55.0**	79.5	88.6	**55**	**31.4**
		Male	1159	79.7	**96.8**	**63.4**	80.9	87.0	**63**	**38.0**
	Coastal regions	No	1654	**82.0**	**96.3**	**63.0**	**90.2**	88.0	**50.5**	34.9
		Yes	763	**73.4**	**91.7**	**50.5**	**58.5**	87.4	**60.1**	33.7
2015	Total	Total	2555	87.9	98.4	59.3	79.7	95.1	58.8	21.5
	Region	Urban	1613	**86.9**	**97.6**	**57.3**	**81.0**	**94.0**	62.4	**23.8**
		Rural	942	**89.7**	**99.7**	**62.6**	**77.6**	**96.8**	69	**17.6**
	Age, year	18~44	1115	**89.0**	98.2	**54.9**	78.7	**97.3**	60.1	**18.6**
		45~59	885	**88.5**	98.8	**59.4**	80.6	**96.2**	55.3	**22.1**
		≥60	555	**84.9**	98.0	**67.9**	80.5	**88.8**	66.3	**26.5**
	Gender	Female	1317	88.7	**97.5**	**53.8**	79.3	94.6	54.3	20.6
		Male	1238	87.1	**99.3**	**65.2**	80.1	95.6	64.3	22.5
	Coastal regions	No	1290	87.7	**98.9**	**54.8**	**70.2**	**96.0**	**56**	**18.4**
		Yes	1265	88.1	**97.8**	**63.9**	**89.5**	**94.2**	**62.9**	**24.7**
2022	Total	Total	5609	68.6	96.5	62.9	73.0	91.0	59.3	36.4
	Region	Urban	3036	**61.2**	**97.0**	**59.3**	**64.2**	**90.3**	67.1	**41.6**
		Rural	2573	**77.2**	**95.9**	**67.1**	**83.4**	**91.8**	69.8	**30.2**
	Age, year	18~44	1901	**70.7**	**96.8**	55.0	74.2	90.1	63.8	**31.2**
		45~59	1897	**64.9**	**97.0**	69.8	73.6	91.3	55	**42.7**
		≥60	1811	**70.1**	**95.6**	63.8	71.0	91.7	67.9	**35.2**
	Gender	Female	3040	**70.0**	**95.5**	**58.6**	**71.9**	90.6	58.6	37.1
		Male	2569	**66.9**	**97.7**	**67.9**	**74.3**	91.6	61	35.5
	Coastal regions	No	3158	**61.2**	**98.6**	**64.3**	**69.0**	**90.2**	64.3	37.2
		Yes	2451	**78.0**	**93.7**	**61.0**	**78.1**	**92.0**	56.5	35.4
2023	Total	Total	17071	61.5	96.6	56.5	68.9	92.8	56.9	47.7
	Region	Urban	10045	**65.3**	96.8	56.3	**74.4**	**93.3**	**56.3**	**45.9**
		Rural	7026	**56.1**	96.3	56.9	**61.1**	**92.1**	**64.5**	**50.3**
	Age, year	18~44	7323	**62.8**	96.9	**49.6**	69.3	92.5	**57.7**	**47.1**
		45~59	5781	**60.1**	96.5	**64.5**	67.8	92.9	**49.6**	**50.0**
		≥60	3967	**61.2**	96.3	**57.7**	69.9	93.3	**62.8**	**45.4**
	Gender	Female	8857	**63.0**	**95.6**	**50.7**	**66.8**	**92.0**	**50.7**	**49.5**
		Male	8214	**59.9**	**97.7**	**62.8**	**71.2**	**93.7**	**56.6**	**45.7**
	Coastal regions	No	9733	**68.1**	96.8	56.6	**80.3**	**93.2**	**56.4**	**49.3**
		Yes	7338	**52.8**	96.4	56.4	**53.8**	**92.2**	**60**	**45.5**
2024	Total	Total	16726	53.2	97.5	60.0	69.1	93.1	58.4	47.3
	Region	Urban	9842	**50.9**	97.6	**61.1**	**73.9**	**93.6**	61.1	**46.6**
		Rural	6884	**56.5**	97.3	**58.4**	**62.4**	**92.3**	68.6	**48.3**
	Age, year	18~44	7192	**54.8**	97.6	**53.8**	69.3	**92.4**	**59.2**	**45.7**
		45~59	5523	**51.9**	97.2	**68.6**	69.1	**93.8**	**53.8**	**48.8**
		≥60	4011	**52.2**	97.6	**59.2**	69.0	**93.2**	**66**	**48.1**
	Gender	Female	8828	**55.1**	**96.5**	**54.5**	**67.3**	**92.7**	**54.5**	**48.8**
		Male	7898	**51.1**	**98.5**	**66.0**	**71.3**	**93.6**	**60.5**	**45.6**
	Coastal regions	No	9458	**50.0**	97.5	60.5	**79.0**	93.0	**76.4**	47.6
		Yes	7268	**57.4**	97.5	59.3	**56.4**	93.1	**74.8**	46.9

Note: Values in bold indicate statistically significant differences between subgroups within the same surveillance cycle.

## Data Availability

The data presented in this study are available on request from the corresponding author due to privacy and ethical concerns in this survey.

## Abbreviations

The following abbreviations are used in this manuscript:

CVDs	Cardiovascular diseases
PAF	Population attributable fraction
RR	Relative risk
SSBs	Sugar-sweetened beverages
3 d–24 h	Three days consecutive 24 h recalls
95%CI	95% Confidence Interval

## Appendix A

**Table A1:** Relative Risks (RRs) of cardiovascular disease mortality associated with dietary factors.

Dietary Factor	Definition of Suboptimal Exposure	Exposure Contrast/ Reference Category	Outcome	RR (95% CI)	Data Source	Population/ Region
Fruit	<300 g/day	Low fruit intake vs. reference intake of ≥300 g/day	CVD mortality	1.17 (1.12–1.22)	Fruit and vegetable consumption and risk of cardiovascular disease: A meta-analysis of prospective cohort studies	Including Asia
Vegetables	<300 g/day	Low vegetable intake vs. reference intake of ≥300 g/day	CVD mortality	1.16 (1.11–1.22)	Fruit and vegetable consumption and risk of cardiovascular disease: A meta-analysis of prospective cohort studies	Including Asia
Aquatic products	<50 g/day	Low fish/aquatic-product intake vs. reference intake of ≥50 g/day	CVD outcome specified in source	1.07 (1.02–1.14)	Fish Consumption and the Risk of Chronic Disease: Fish Intake in Relation to Fatal and Non-Fatal Cardiovascular Risk: A Systematic Review and Meta-Analysis of Cohort Studies.	Including Asia
Condiment salt	≥5 g/day	High salt intake vs. lower/reference salt intake	CVD/stroke outcome specified in source	1.17 (1.02–1.34)	Salt intake, stroke, and cardiovascular disease: meta-analysis of prospective studies	Including Asia
Nuts	<20 g/day	Low nut intake vs. reference intake of ≥20 g/day	CVD mortality	1.23 (1.12–1.35)	Nut consumption and risk of cardiovascular disease, total cancer, all-cause and cause-specific mortality: a systematic review and dose-response meta-analysis of prospective studies	Including Asia
Red meat	≥100 g/day	High red-meat intake vs. lower/reference intake	CVD outcome specified in source	1.11 (1.05–1.16)	Red meat consumption, cardiovascular diseases, and diabetes: a systematic review and meta-analysis	Asia
Processed meat	≥50 g/day	High processed-meat intake vs. lower/reference intake	CVD outcome specified in source	1.26 (1.18–1.35)	Red meat consumption, cardiovascular diseases, and diabetes: a systematic review and meta-analysis	Asia
SSBs	≥50 g/day	High SSB intake vs. lower/reference intake	CVD outcome specified in source	1.08 (1.04–1.13)	Intake of Sugar-Sweetened and Low-Calorie Sweetened Beverages and Risk of Cardiovascular Disease-A Meta-Analysis and Systematic Review	Including Asia
Whole grains	<35 g/day	Low whole-grain intake vs. reference intake of ≥35 g/day	CVD mortality	1.22 (1.18–1.28)	Whole-grain intake and total, cardiovascular, and cancer mortality: a systematic review and meta-analysis of prospective studies	Including Asia
Omega-3 fatty acids	<0.175 g/day	Low omega-3 intake vs. higher/reference intake	Mortality outcome specified in source	1.26 (1.01–1.59)	Intake of Long-Chain ω-3 Fatty Acids From Diet and Supplements in Relation to Mortality	America
